# Segmental resection vs. partial resection on treating solid multicystic ameloblastomas of the jaws – recurrence rates: A systematic review and meta-analysis

**DOI:** 10.4317/jced.60502

**Published:** 2023-07-01

**Authors:** Rafael Netto, Mariela Peralta-Mamani, Silas-Antonio-Juvencio de Freitas-Filho, Ludimila-Lemes Moura, Cassia-Maria-Fischer Rubira, Izabel-Regina-Fischer Rubira-Bullen

**Affiliations:** 1 Department of Surgery, Stomatology, Pathology and Radiology, Bauru School of Dentistry, University of São Paulo, SP, Brazil; 2 Faculdade do Centro Oeste Paulista - FACOP, Piratininga, São Paulo, Brazil; 3 School of Dentistry, University Center - UNIFAE, São João da Boa Vista, SP, Brazil

## Abstract

**Background:**

The aim of the present study was to compare the recurrence rates of solid multicystic ameloblastomas after segmental resection or marginal resection.

**Material and Methods:**

PubMed, ScienceDirect, Web of Science, Scopus, Embase were searched for studies published up to July 2022. The gray literature was also searched. Meta-analysis was performed using OpenMeta Software, *p*< 0.05 considered significant.

**Results:**

Among the search, 8 studies met all eligibility criteria. The group that underwent marginal resection was 1.1 times more likely to present recurrence of the lesion compared to the group that underwent segmental resection. There was no statistically significant difference between the two groups (segmental resection and marginal resection) in all eight studies regarding reducing ND (95% Confidence interval, 0.339 – 3.705; heterogeneity: Q value= 3.105; I2= 0%).

**Conclusions:**

The results showed that there was no statistically significant difference between segmental and marginal resection for the treatment of solid multicystic ameloblastomas; however, prospective studies with more rigorous methodological procedures are needed to better compare the surgical techniques.

** Key words:**Ameloblastoma, solid multicystic ameloblastoma, treatment, recurrence.

## Introduction

Odontogenic tumors are among the most prevalent bone alterations of the jaws (1) *et al*., 2022). Ameloblastoma is one of the most common benign odontogenic tumors, with a prevalence between 75.5% and 32.9% (2,3). Despite being among the benign odontogenic tumors, ameloblastoma can present locally aggressive behavior (4).

The 5th Edition of the World Health Organization (WHO) Classification of Head and Neck Tumors in 2022 distinguishes five types of ameloblastoma: extraosseous or peripheral, unicystic, conventional (solid multicystic), a new adenoid entity, and metastasizing (5). Conventional ameloblastoma is the most common type ranging from 57% to 63.8. There is no clear sex predilection, although some studies show a slightly higher number of affected men. The population between the second and fourth decade of life is the most affected (4,6).

The treatment of ameloblastoma can be conservative (marsupialization, enucleation, curettage), which is generally used in cases of unicystic ameloblastoma; or radical, which corresponds to marginal resection (also called partial) and segmental resection, both used in cases of conventional ameloblastoma (7).

Recurrence of ameloblastoma is high, especially in cases treated conservatively (6). However, radical treatment involves a greater number of postoperative complications, in addition to a decrease in quality of life because it affects regions that are fundamental for the aesthetics and function of the stomatognathic system (4,8).

Thus, the aim of this study was to answer the following question through a systematic review: “In patients with solid multicystic ameloblastomas, what is the recurrence rate of segmental resection compared to marginal resection?”.

 

## Material and Methods

This systematic review was conducted in accordance with the PRISMA guidelines (Preferred Reporting Items for Systematic Reviews and Meta-Analyses) (9).

In order to establish the PICO strategy criteria, all studies had to answer the following question: “In patients with solid multicystic ameloblastomas, what is the recurrence rate of segmental resection compared to marginal resection?”. Participants (P) were individuals with solid multicystic ameloblastoma; the intervention (I) was segmental or partial resection; and the control (C) was partial resection; outcomes (O) recurrence rates.

-Protocol and registration 

The protocol of this systematic review was registered in the PROSPERO database (CRD42018084812) and is available at http://www.crd.york.ac.uk/PROSPERO/.

-Eligibility criteria

To be included in the study sample, publications had to meet the following selection criteria: 1. studies comparing two treatments for solid multicystic ameloblastoma (segmental resection vs. partial resection); 2. cross-sectional and retrospective studies. Descriptive literature reviews, clinical reports and series of clinical reports without highlighting treatments and their comparisons, as well as those that evaluated only unicystic ameloblastomas were excluded.

-Information sources

A complete literature review was performed to identify studies comparing the segmental resection with the partial resection about recurrence rates in patients with solid multicystic ameloblastoma. The identification of studies was based on a search strategy for each of the following electronic databases: PubMed, ScienceDirect, Web of Science, Scopus, Embase. In addition, gray literature (Google Scholar) and manual search of the reference list were also searched.

-Search strategy

A descriptive search strategy was structured with Boolean operators (AND/OR/NOT) and designed to determine all relevant studies published up to July 12, 2022. There was no restriction by year of publication. The following descriptors were used: (Solid ameloblastoma OR Ameloblastoma) AND (Neoplasm Recurrence, Local OR Recurrence OR Recrudescence OR Relapse) AND (Treatment Outcome OR Treatment Effectiveness OR Rehabilitation Outcome) AND (Jaw Neoplasms OR Cancer of Jaw OR Jaw Cancer). Also, the grey literature was searched in order to include any additional paper that might meet the eligibility criteria.

-Data collection process

The articles were imported into the reference manager EndNote Web for organizing and excluding duplicates from different databases.

According to the research strategies specified above, the articles selected were passed through two of the authors (RN and MPM), who read the title and abstract independently, making a first selection within the inclusion and exclusion criteria. Afterwards, the selected articles were to be read completely for a final decision about which articles will remain in the research. A third author was responsible for resolving the discrepancy (IRFRB).

-Data items

A standardized form was used to extract data from the studies included in this systematic review and summarize results. Data extraction was performed by two independent examiners (RN and SAJFF), obtaining the following characteristics: author, year of publication, country of origin of the study, number of patients of interest, interval, type of treatment, relapse and follow-up in months. The details of each study are presented in Table 1.

**Table 1:**
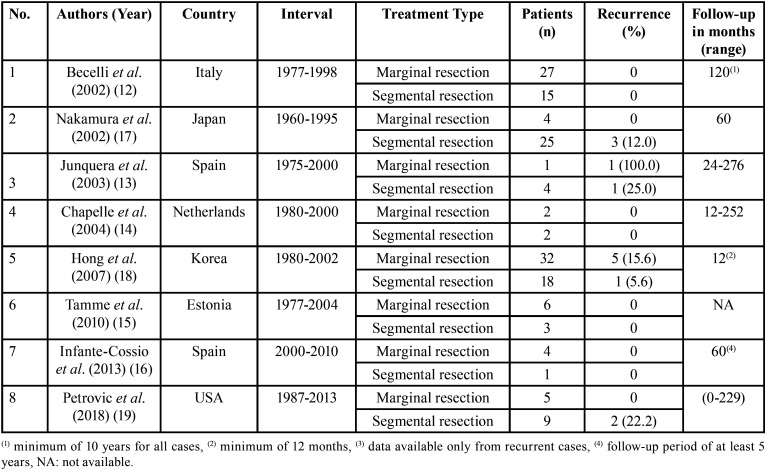
Summary of studies included in the review.

-Risk of bias in the individual studies

The analysis of the risk of bias in individual studies was assessed using the JBI Critical Appraisal Checklist for Case Series by Faculty of Health and Medical Sciences at the University of Adelaide, South Australia (2020) (10). This tool was used due to the inclusion of patients with a specific disease and some of the evaluated items are directly related to the risk of bias, while others are related to the guarantee of the cases presented and adequate statistical analysis. Two independent reviewers (SAJFF and LLM) performed the evaluations of the selected studies. Cases of disagreement during the evaluation of the studies were resolved by consensus reunions.

-Effect measures

The data collected on the recurrence rate of partial resection and segmental resection as treatment for solid multicystic ameloblastoma were case frequency and percentage.

-Synthesis methods 

The quantitative analysis was performed using a meta-analysis using the OpenMeta Software [Analyst], considering the random effect model (11), with a confidence interval of 95%, significance level of 5%, correction factor 0.5. Heterogeneity was explored by performing sensitivity analysis.

## Results

-Study selection

**Figure 1:**
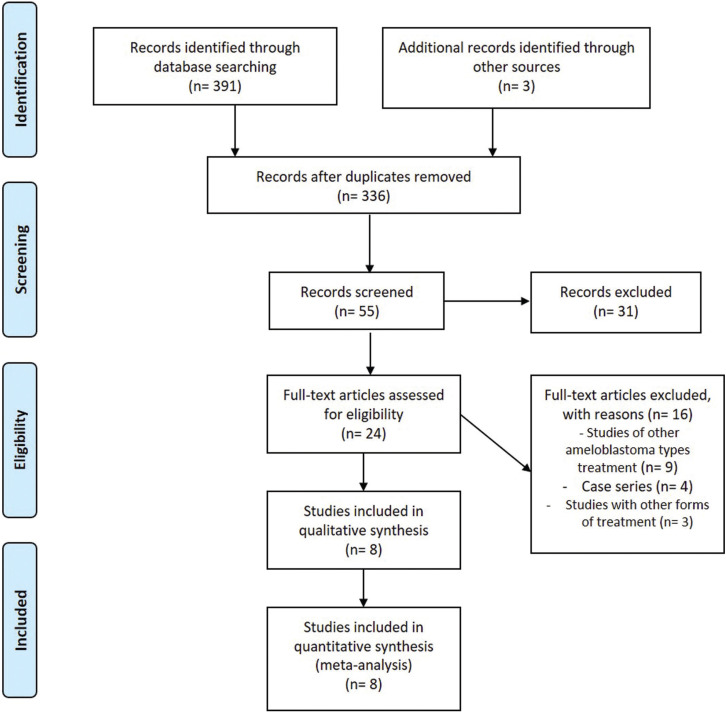
Flowchart for the identification of included and excluded studies.

Using EndNote Web to remove duplicate records, in all, 336 records were identified during the search. Then, 55 articles were retained for title and abstract reading. Of these, 31 were excluded. Twenty-four articles were selected for full-text reading, and only eight were finally selected (12-19). The other 16 studies were excluded (20-35) for the following reasons: studies with other types of ameloblastoma treatment (n= 9), case series (n= 4) and studies with other forms of treatment (n= 3). In total, eight studies were selected (Fig. 1).

-Study characteristics

Of the eight studies selected five are European (12-16), two are Asian (17,18) and one is North American (19). All studies presented recurrence data. Only one study did not detail the follow-up time of patients (15).

A total of 158 patients were included in this review. In these studies, all patients received the histopathological diagnosis of solid multicystic ameloblastoma. In all studies, demographic and clinical data were quite detailed; however, they also provided information on other types of ameloblastomas and forms of treatment, which are not of interest in this review and for this reason are not detailed in this review.

-Risk of bias in studies

In the present study, the JBI Critical Assessment Tool for Case Series Studies was applied to assess the risk of bias in the eight selected studies. This tool includes 10 questions that address the internal validity of case series studies. The individual result of each study for each question is detailed in Table 2.

**Table 2:**
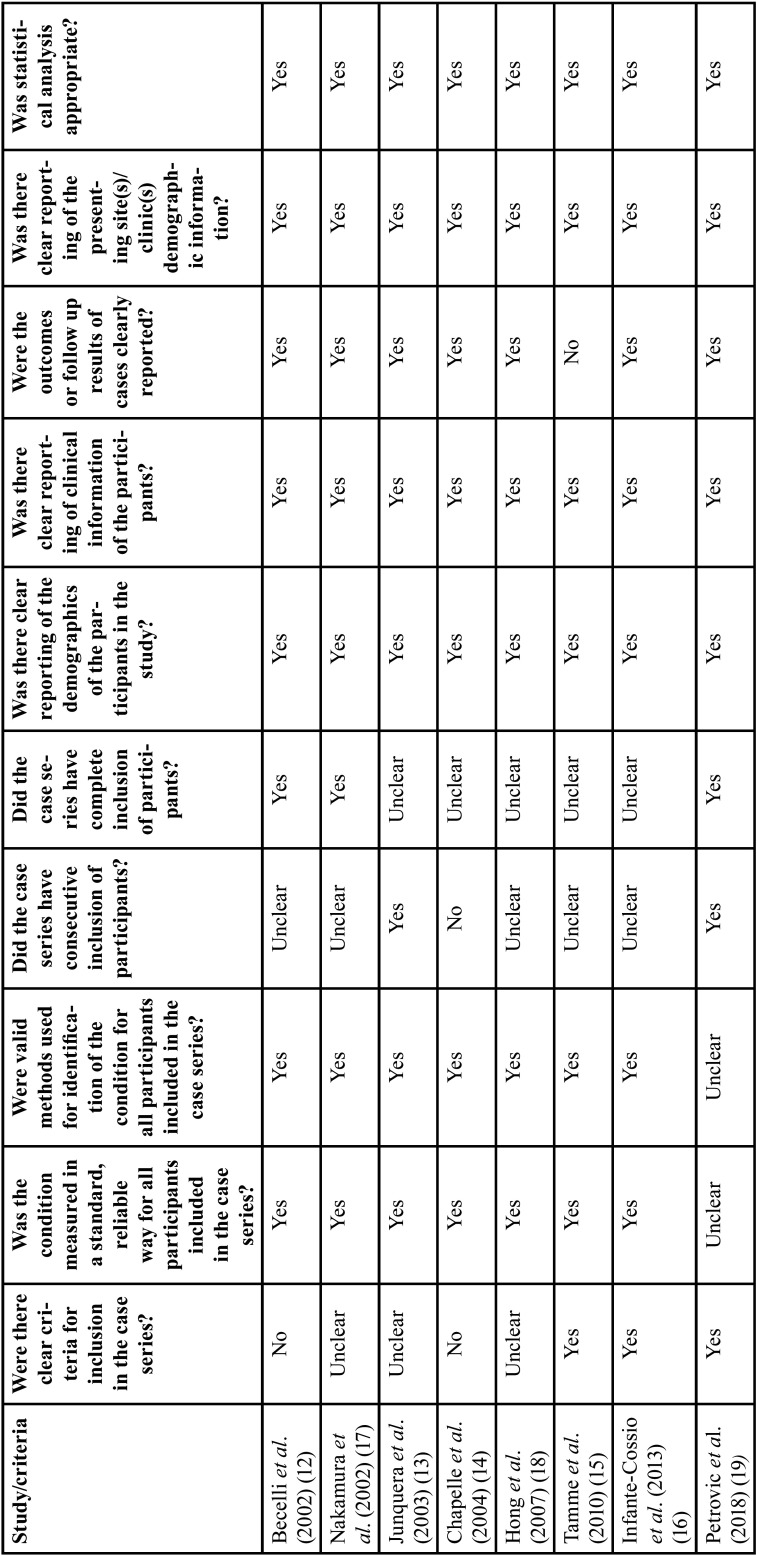
Quality assessment of individual study.

Regarding the inclusion criteria, three studies clearly presented it (15,16,19), two studies did not present it (12,14) and three others were unclear (13,17,18). In only one study, standard and reliably measured conditions and valid methods for identifying conditions for all included participants were unclear (19).

Regarding the consecutive inclusion of patients in the studies, most studies were unclear (12,15-18), two said they had done it (13,19) and one denied it (14). In five studies the complete inclusion of patients was unclear (13-16,18), and in the other three there was complete inclusion (12,17,19).

In the eight selected studies, clear reports of the demographics and clinical information of the participants were presented (12-19). Only one study did not clearly present patient follow-up results (15). In all papers, there were clear reports of the demographic information of the presenting site(s)/clinic(s) (12-19). Furthermore, statistical analysis was appropriate in all these studies (12-19).

-Results of individual studies

The study of Hong *et al*. (18) had the highest number of patients who received marginal or segmental resection as treatment and only six out of fifty patients had recurrence. In four studies, the number of patients included for comparison between treatments was less than 10 (13-16). In four studies, none of the 60 patients presented recurrence after partial or segmental resection treatments (12,14-16).

The recurrence rate among patients with solid multicystic ameloblastoma who underwent partial resection ranged from 15.6 to 100% (13,15). While the recurrence rate among those treated with segmental resection ranged from 5.6% to 25.0% (13,17-19).

-Results of synthesis

**Figure 2:**
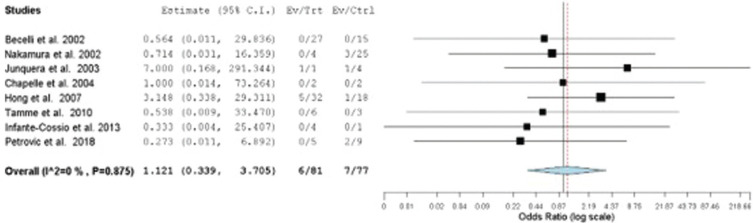
Forest graph of solid multicystic ameloblastoma recurrence rate comparison of patients treated with marginal and partial resection.

A meta-analysis on the recurrence rate of solid multicystic ameloblastoma treated by marginal resection compared to partial resection was performed with eight studies (12-19). The group that underwent marginal resection was 1.1 times more likely to present recurrence of the lesion compared to the group that underwent segmental resection. However, there was no statistically significant difference between groups (*p*= 0.851) (Confidence interval 95%, 0.339 – 3.705; Heterogeneity: Q value 3.105; I2 0%; Tau2 0.000; *p-value* 0.875. N Marginal resection= 81, N Segmental resection = 77) (Fig. 2).

To explore heterogeneity through sensitivity analysis, only studies with samples larger than 10 patients were considered. The result also showed that there were no differences between the rates of recurrence of the lesion in the group that underwent marginal resection and segmental resection. (*p*= 0.626; Confidence interval 95%, 0.248 – 4.696; Heterogeneity: Q value 1.751; I2 0%; Tau2 0.000; *p-value* 0.626. N Marginal resection = 68, N Segmental resection = 67). Therefore, for this systematic review, the forest chart with the 8 included studies was considered.

## Discussion

Despite being considered a benign pathological entity, ameloblastoma is a locally invasive tumor, with potential chances of recurrence after surgical removal (12-19). Multicystic solid ameloblastomas, currently called conventional, require a more radical treatment when compared to unicystic ameloblastomas because they present higher recurrence rates (5,7). Despite the treatment recommendations according to the type of ameloblastoma, it is noted that some services have shown a wide variety of surgical techniques (7).

In the present study, we performed a systematic review and meta-analysis to assess recurrence rates between segmental and marginal resection in solid multicystic ameloblastomas. After selecting the articles, as shown in Fig. 1, eight studies met the eligibility criteria. All included studies were retrospective and the evidence is limited. The information necessary to answer the question of our systematic review was extracted from these studies. These works also presented information on unicystic amelobalstomas, which were ignored. This explains the absence of demographic and clinical information in Table 1. It was possible to extract from these studies information on treatment and recurrence rates of multicystic solid ameloblastomas.

Recurrence rates for solid multicystic ameloblastomas after segmental resection ranged from 5.6% to 25.0% (13,17-19). For those who were treated with marginal resection these values ranged from 15.6% to 100% (13,18). However, in this study, which showed 100% recurrence, only one patient underwent marginal resection (13). Interestingly, two studies showed recurrence of solid multicystic ameloblastoma in patients treated with segmental resection and no recurrence among those who underwent marginal resection (17,19). On the other hand, Hendra *et al*. (2019), noted that radical treatment was more satisfactory treatment than conversational treatment for solid multicystic ameloblastomas and unicystic ameloblastomas. In patients treated by Hong *et al*. (18), the recurrence rate for cases treated with marginal resection was almost three times higher compared to those who underwent segmental resection.

The forest plot shows that there was no difference between marginal resection and segmental resection treatments for solid multicystic ameloblastoma. However, it is important to consider the limited number of studies and the variable sample size between populations. There was heterogeneity of the studies due to the methodology of each one of them, highlighting that the follow-up time was different among the various studies included. Furthermore, the included studies had in their sample different treatments for various types of ameloblastomas (12-19). Since the objective of this study was to compare the recurrence rate only in the treatment of solid multicystic ameloblastoma, only these cases were considered, reducing the sample number of patients (12-19).

Our study has some limitations, among them: the time between surgery and recurrence; follow-up time between patients was not uniform; reduced number of participants in some studies; and, limitation in extracting demographic and clinical data. However, our systematic review is the first to compare the recurrence rates of solid multicystic ameloblastomas after segmental and marginal resection.

The performance of prospective studies with more demanding methodological procedures in their stages, such as detailing demographic and clinical information, describing surgical protocols and improving the follow-up time of patients, evidencing the moment of recurrence.

## Conclusions

Within the limitations of the systematic review, the results showed that there was no statistically significant difference between segmental and marginal resection for the treatment of solid multicystic ameloblastomas. However, prospective studies with more rigorous methodological procedures are needed to better compare the surgical techniques.
